# Prognostic assessment of breast carcinoma submitted to neoadjuvant chemotherapy with pathological non-complete response

**DOI:** 10.1186/s12885-019-5812-0

**Published:** 2019-06-17

**Authors:** Uanderson Resende, César Cabello, Susana Oliveira Botelho Ramalho, Luiz Carlos Zeferino

**Affiliations:** Division of Gynecological and Mammary Oncology, Woman’s Hospital Dr José Aristodemo Pinotti (CAISM) of State University of Campinas (UNICAMP), Rua Alexander Fleming 101, Campinas, São Paulo 13083-083 Brazil

**Keywords:** Breast, Carcinoma, Neoadjuvant therapy, Prognosis, Disease-free survival

## Abstract

**Background:**

Breast cancer with pathological non-complete response (non-pCR) after neoadjuvant chemotherapy (NAC) has a worse prognosis. Despite Neo-Bioscore has been validated as an independent prognostic model for breast cancer submitted to NAC, non-pCR carcinoma was not assessed in this setting.

**Methods:**

This is a retrospective trial that included women with localized breast cancer who underwent NAC and had non-pCR carcinoma in surgical specimen between 01/01/2013 to 12/31/2015 with a three-year follow-up. Survival analysis was performed by Kaplan-Meier estimator and hazard ratio (HR) set by log-rank test for the primary and secondary endpoints, respectively Disease-Free Survival (DFS) and Overall Survival (OS). According to Neo-Bioscore, the proposed prognostic model named Clustered Neo-Bioscore was classified into low (0–3), low-intermediate (4–5), high-intermediate (6) and high (7) risk. The prognostic accuracy for recurrence risk was assessed by time-dependent receiver operating characteristic (time-ROC) methodology. Multivariate Cox regression assessed the menopausal status, histological grade, Ki-67, estrogen receptor, HER2, tumor subtype, pathological and clinical stages. Confidence interval at 95% (CI95%) and statistical significance at set 2-sided *p*-value less than 0.05 were adopted.

**Results:**

Among the 310 women enrolled, 267 patients (86.2%) had non-pCR carcinoma presenting size T3/T4 (63.3%), node-positive axilla (74.9%), stage III (62.9%), Ki-67 ≥ 20% (71.9%) and non-luminal A (78.3%). Non-pCR carcinoma presented worse DFS-3y (HR = 3.88, CI95% = 1.18–11.95) but not OS-3y (HR = 2.73, CI95% = 0.66–11.40). Clustered Neo-Bioscore discerned the recurrence risk for non-pCR carcinoma: low (DFS-3y = 0.86; baseline), low-intermediate (DFS-3y = 0.70; HR = 2.61), high-intermediate (DFS-3y = 0.13, HR = 14.05), and high (DFS-3y = not achieved; HR = 22.19). The prognostic accuracy was similar between Clustered Neo-Bioscore and Neo-Bioscore (0.76 vs 0.78, *p* > 0.05). Triple-negative subtype (HR = 3.6, CI95% = 1.19–10.92) and pathological stages II (HR = 5.35, CI95% = 1.19–24.01) and III (HR = 6.56, CI95% = 1.29–33.32) were prognoses for low-intermediate risk, whereas pathological stage III (HR = 13.0, CI95% = 1.60–106.10) was prognosis for low risk.

**Conclusions:**

Clustered Neo-Bioscore represents a novel prognostic model of non-pCR carcinoma undergoing NAC with a more simplified and appropriate score pattern in the assessment of prognostic factors.

**Electronic supplementary material:**

The online version of this article (10.1186/s12885-019-5812-0) contains supplementary material, which is available to authorized users.

## Background

Breast cancer is the most common malignant tumor in women worldwide with about 22% of all new cancers in 2015 [1]. In recent years, new data on the molecular heterogeneity of breast carcinoma have led to a greater understanding of the diversity of prognosis [2]. Neoadjuvant chemotherapy (NAC) is one of the main therapeutic modalities in the treatment of high-risk breast cancer [3] due to tumor downstaging with increased rate of conservative breast surgery [4], better model of clinical research to evaluate the performance of biomarkers and tumor sensitivity to chemotherapeutic agents [5], generally using a smaller sample size and shorter follow-up time [6].

The main prognostic factors of breast cancer are age, clinical performance, menopausal status, tumor stage [7], histological type, tumor differentiation, gene expression signature [8, 9], molecular subtype [10, 11], expression of estrogen (ER) [12, 13] and progesterone receptors (PR) [14], HER2 oncoprotein (HER2) [15] and cellular proliferation index Ki67 (Ki-67) [16–18]. However, these factors alone have limited prognostic power while combining them into an algorithm increases the prognostic power [19]. Neo-Bioscore is currently the leading prognostic model for assessing the recurrence risk in women with breast cancer undergoing NAC. It is calculated from the sum of the scores obtained from the degree of tumor differentiation, ER, HER2, clinical and pathological stages [20–22]. However, Neo-Bioscore has eight recurrence risk scores (0 to 7) whose survival curves presented a non-linear time-to-event distribution among them due to the non-uniform distance among time-to-event occurrences [21, 22].

Breast carcinoma undergoing NAC that did not present a pathological complete response (non-pCR) has a worse prognosis [23–27] and there is little information on its independent prognostic factors [28]. Therefore, it would be appropriate to assess a novel prognostic model that is more simplified to deal with linear stratification of scores for women with non-pCR carcinoma. We propose a novel prognostic model named Clustered Neo-Bioscore from the clustering of similar prognostic scores of Neo-Bioscore which is composed of four scores - low, low-intermediate, high-intermediate and high-risk - whose risk strata are linearly more distanced from each other as to the time-to-event of tumor recurrence in the survival curve. Clustered Neo-Bioscore and Neo-Bioscore were compared for prognostic accuracy. Independent prognostic factors for non-pCR carcinoma were analyzed by Cox regression according to Neo-Bioscore, low and low-intermediate Clustered Neo-Bioscore.

## Methods

### Patients

This is a retrospective cohort that included women with localized breast carcinoma who underwent NAC followed by surgery at the Woman’s Hospital Dr. José Aristodemo Pinotti (CAISM) of the State University of Campinas (UNICAMP), between 01/01/2013 to 12/31/2015. It was approved by the Ethics Committee in Research of the Faculty of Medical Sciences (FCM) of UNICAMP. A total of 345 women were selected but 35 patients were excluded due to neoadjuvant hormonal therapy (25), surgical contraindication (2), clinical comorbidities (2), or metastases diagnosed throughout NAC (6). Therefore, 310 women were enrolled in this analysis.

### Materials

The clinical and biological factors analyzed were menopausal status, tumor size, axilla status, clinical stage, histologic grade, ER, Ki67, HER2 and molecular subtypes according to immunohistochemical findings [18, 29–31]. Pathologic analyzes and immunohistochemical assays of core needle biopsy and surgical specimen were performed in the same institutional laboratory. Menopausal status was defined as the presence of amenorrhea greater than 1 year or serum estradiol levels below 10 pg/ml. The ER expression was classified by Allred scoring into negative, low (2–6) and high (7–8) [32]. The Ki-67 expression was subdivided into low (< 20%), intermediate (20% ≤ Ki67 < 50%) and high (≥ 50%) [33, 34]. Molecular subtypes were luminal A, luminal B, HER2 and triple-negative (TN) based on the results of the immunohistochemistry assay [9, 29–32].

### NAC protocol

Enrolled women received 4 cycles of doxorubicin 60 mg/m^2^ and cyclophosphamide 600 mg/m^2^ at intervals of 3 weeks followed by 4 cycles of paclitaxel 210 mg/m^2^ at intervals 3 weeks (4 AC + 4 T) or 12 cycles of paclitaxel 80 mg/m^2^ weekly (4 AC + 12 T) [35]. About 85% (63/74) of women with HER2 carcinoma also received trastuzumab at 4 mg/kg (initial dose) followed by 2 mg/kg weekly for 23 consecutive weeks plus 9 cycles at 6 mg/kg at intervals 3°weeks [36]. Approximately 1% (9/94) of women with TN carcinoma also received carboplatin AUC = 1.5 weekly associated with paclitaxel [37]. None of the women showed serious adverse effects that required discontinuation of NAC. No one dropped out of NAC due to loss of follow-up or weak treatment compliance. There were dose reductions and/or delays for 52 women by neuropathic (37) and emetic (9) toxicity, surgery anticipation (4) or no clinical response (2). Dose reduction by only 20% (43) and/or one cycle (4) was the majority and therefore without influencing the overall efficacy of NAC. The period between the last cycle of chemotherapy and surgery was 20–30 days. Surgical clips were used to identify the tumor location by mammography in the surgery. The clinical staging by TNM classification was performed by clinical examination, mammography, breast and axillary ultrasound, bone scan computed tomography of chest and abdominal [38, 39]. Supportive care such as antiemetic, blood transfusion and antimicrobial therapy were given according to oncologist’s discretion. The pCR was defined as the absence of invasive carcinoma in surgical breast specimen and micro or macrometastases in removed axillary lymph nodes [24, 27, 36–38].

### Treatment assessment

The principal outcome was Disease-Free Survival (DFS) that was defined from the date of diagnosis until the date of death, locoregional or distant recurrence. The secondary outcome was Overall Survival (OS) that was defined from the date of diagnosis until the date of death. The follow-up time was 37 months. The Neo-Bioscore was calculated from the sum of the scores of the following data: clinical stage (I/IIA: 0 point, IIB/IIIA: 1 point, IIIB/IIIC: 2 points), pathological stage (0/I: 0 point; IIIB: 1 point, IIIC: 2 points), ER (negative: 1 point; positive: 0 point), nuclear grade (I/II: 0 point; III: 1 point) and HER2 (negative: 1 point; positive: 0 point) [19–22]. The Clustered Neo-Bioscore was calculated from Neo-Bioscore by grouping prognostic scores similar into each other to obtain four very different prognostic categories: low (0, 1, 2 or 3), low-intermediate (4 or 5), high-intermediate (6) and high (7). This configuration of categories presented greater homogeneity within them and heterogeneity among them leading to a greater time-to-event distance for tumor recurrence among the categories.

### Statistical analysis

Statistical analysis was performed by SPSS 22.0 software (version 22; SPSS Inc). Categorical variables were analyzed with Pearson’s chi-square test and Kendal’s Tau for multinomial regression according to pathological response and Neo-Bioscore. The Kaplan-Meier estimator was used to obtain the time-to-event for recurrence tumor of pCR and non-pCR carcinomas according to Neo-Bioscore and Clustered Neo-Bioscore, survival curves for a 3-year follow-up and the hazard ratio (HR) between pCR and non-pCR carcinomas for DFS-3y and OS-3y. The log-rank test was used to compare the prognosis among the risk scores of the pathological stage (I, II and III), Neo-Bioscore (0 to 7) and Clustered Neo-Bioscore (low, low-intermediate, high-intermediate and high). The mean time to first relapse (mTFR) and the respective HR among the risk categories of Clustered Neo-Bioscore were calculated by Kaplan-Meier, while the statistical significance was defined by the log-rank test. The multivariate analysis by Cox proportional hazards regression was performed to define the independent prognostic factors of non-pCR carcinoma according to Neo-Bioscore, low and low-intermediate Clustered-Neo-Bioscore. The Cox regression for intermediate-high and high risk was not performed because of the limited number of patients in these groups [40, 41]. The prognostic accuracy for recurrence risk among the pathological stage, Neo-Bioscore and Clustered Neo-Bioscore was assessed from the area under the curve (AUC) by time-dependent receiver operating characteristic (time-ROC) methodology. The confidence interval at 95% (CI95%) was adopted and statistical significance was at set 2-sided *p*-value less than 0.05 (*p* <  0.05) [42].

## Results

### Patient characteristics

Women with high-risk breast cancer prevailed with size T3/T4 (64%), stage III (62%), positive axilla (74%), Ki-67 ≥ 20% (74%) and non-Luminal A (81%). The pCR carcinoma was about 13.8% (43/310) of the total with histological grade III (62.8%), negative ER (74.4%), Ki-67 ≥ 20% (88.4%), HER2 and TN (93%) subtypes, T3/T4 size (69.8%), N1 axilla (48.8%) and stage III (58.1%). Otherwise, the non-pCR carcinoma presented similar clinical characteristics to pCR carcinoma with size T3/T4 (63.3%), axilla N1 (43.8%) and stage III (62.9%) but different biological factors with histological grade I/II (53.9%), ER Allred 7–8 (49.4%), Ki-67 < 50% (71.5%) and Luminal B (30.3%) (Table 1).

**Table 1:** Clinical and biological characteristics of breast carcinoma according to pathological response and Neo-Bioscore

Characteristics	n	Pathological response		Neo-Bioscore							
		pCR^a^	non-pCR	0	1	2	3	4	5	6	7
	310 (100%)	43 (13.8%)	267 (86.2%)	1 (0.3%)	18 (5.8%)	47 (15.2%)	84 (27.1%)	83 (26.8%)	49 (15.8%)	23 (7.4%)	5 (1.6%)
Menopausal status											
Premenopausal	148 (48%)	21 (48.8%)	127 (47.6%)	0 (0%)	10 (55.6%)	31 (66%)	42 (50.0%)	33 (39.8%)	22 (44.9%)	8 (34.8%)	2 (40%)
Postmenopausal	162 (52%)	22 (51.1%)	140 (52.4%)	1 (100%)	8 (44.4%)	16 (34%)	42 (50.0%)	50 (60.2%)	27 (55.1%)	15 (65.2%)	3 (60%)
Histological grade											
I/II	160 (52%)	16 (37.2%)	144 (53.9%)	1 (100%)	17 (94.4%)	36 (76.6%)	61 (72.6%)	34 (41.0%)	11 (22.4%)	0 (0%)	0 (0%)
III	150 (48%)	27 (62.8%)	123 (46.1%)	0 (0%)	1 (5.6%)	11 (23.4%)	23 (27.4%)	49 (59.0%)	38 (77.6%)	23 (100%)	5 (100%)
Estrogen receptor											
Negative	131 (42%)	32 (74.4%)	99 (37.1%)	0 (0%)	2 (11.1%)	10 (21.3%)	31 (36.9%)	43 (51.8%)	24 (49%)	16 (69.6%)	5 (100%)
Allred 2–6 (< 70%)	40 (13%)	4 (9.3%)	36 (13.5%)	1 (100%)	4 (22.2%)	7 (14.9%)	11 (13.1%)	8 (9.6%)	8 (16.3%)	1 (4.3%)	0 (0%)
Allred 7–8 (≥70%)	139 (45%)	7 (16.3%)	132 (49.4%)	0 (0%)	12 (66.7%)	30 (63.8%)	42 (50.0%)	32 (38.6%)	17 (34.7%)	6 (26.1%)	0 (0%)
Ki-67											
< 20%	80 (26%)	5 (11.6%)	75 (28.1%)	0 (0%)	6 (33.3%)	13 (27.7%)	27 (32.1%)	20 (24.1%)	12 (24.5%)	2 (8.7%)	0 (0%)
20–50%	134 (43%)	18 (41.9%)	116 (43.4%)	0 (0%)	8 (44.5%)	22 (46.8%)	38 (45.2%)	35 (42.2%)	17 (34.7%)	12 (52.2%)	2 (40%)
≥ 50%	96 (31%)	20 (46.5%)	76 (28.5%)	1 (100%)	4 (22.2%)	12 (25.5%)	19 (22.7%)	28 (33.7%)	20 (40.8%)	9 (39.1%)	3 (60%)
HER2											
Negative	236 (76%)	24 (55.8%)	212 (79.4%)	0 (0%)	10 (55.6%)	28 (59.6%)	62 (73.8%)	66 (79.5%)	45 (91.8%)	20 (87.0%)	5 (100%)
Positive	74 (24%)	19 (44.2%)	55 (20.6%)	1 (100%)	8 (44.4%)	19 (40.4%)	22 (26.2%)	17 (20.5%)	4 (8.2%)	3 (13.0%)	0 (0%)
Subtype											
A	59 (19%)	1 (2.3%)	58 (21.7%)	0 (0%)	5 (27.8%)	9 (19.1%)	18 (21.4%)	16 (19.3%)	10 (20.4%)	1 (4.3%)	0 (0%)
B	83 (27%)	2 (4.7%)	81 (30.3%)	0 (0%)	5 (27.8%)	14 (29.8%)	20 (23.8%)	20 (24.1%)	16 (32.7%)	8 (34.8%)	0 (0%)
HER2	74 (24%)	19 (44.2%)	55 (20.6%)	1 (100%)	8 (40.4%)	19 (40.5%)	22 (26.2%)	17 (20.5%)	4 (8.2%)	3 (13%)	0 (0%)
TN	94 (30%)	21 (48.8%)	73 (27.4%)	0 (0%)	0 (0%)	5 (10.6%)	24 (28.6%)	30 (36.1%)	19 (38.7%)	11 (47.9%)	5 (100%)
Size											
T1/T2	111 (36%)	13 (30.2%)	98 (36.7%)	1 (100%)	17 (94.4%)	28 (59.6%)	42 (50.0%)	21 (25.3%)	2 (4.1%)	0 (0%)	0 (0%)
T3/T4	199 (64%)	30 (69.8%)	169 (63.3%)	0 (0%)	1 (5.6%)	19 (40.4%)	42 (50.0%)	62 (74.7%)	47 (95.9%)	23 (100%)	5 (100%)
Axilla											
Negative	82 (26%)	15 (34.9%)	67 (25.1%)	1 (100%)	12 (66.7%)	24 (51.1%)	24 (28.6%)	11 (13.3%)	9 (18.4%)	1 (4.3%)	0 (0%)
N1	138 (45%)	21 (48.8%)	117 (43.8%)	0 (0%)	5 (27.8%)	19 (40.4%)	37 (44.0%)	45 (54.2%)	22 (44.9%)	8 (34.8%)	2 (40%)
N2/N3	90 (29%)	7 (16.3%)	83 (31.1%)	0 (0%)	1 (5.6%)	4 (8.5%)	23 (27.4%)	27 (32.5%)	18 (36.7%)	14 (60.9%)	3 (60%)
Stage											
I/II	117 (38%)	18 (41.9%)	99 (37.1%)	1 (100%)	16 (88.9%)	32 (68.1%)	42 (50.0%)	21 (25.3%)	5 (10.2%)	0 (0%)	0 (0%)
III	193 (62%)	25 (58.1%)	168 (62.9%)	0 (0%)	2 (11.1%)	15 (31.9%)	42 (50.0%)	62 (74.7%)	44 (89.8%)	23 (100%)	5 (100%)

^a^*pCR* pathological complete response

Most of the carcinomas had Neo-Bioscore ≤5 (91.0%; 282/310) with histological grade I/II (56.7%; 160/282), positive ER (61.0%; 172/282), Ki-67 < 50% (70.2%; 198/282), negative HER2 (74.8%; 211/282), non-TN subtype (72.3%; 204/282), size T3/T4 (60.6%; 171/282), node-positive axilla (71.2%; 201/282) and stage III (62.1%; 175/282). Carcinomas classified as Neo-Bioscore = 0–3 (48.4%; 150/310) prevailed in premenopausal status (55.3; 83/150), histological grade I/II (76.7%; 115/150), ER Allred 7–8 (56.0%; 84/150), Ki-67 < 50% (64.0%; 114/150), negative HER2 (66.7%; 100/150), non-TN subtype (80.7%; 121/150), size T1/T2 (58.7%; 88/150), node-positive axilla (59.3%; 89/150) and stage I/II (60.7%; 91/150). Carcinomas classified as Neo-Bioscore = 4–5 (42.6%; 132/310) prevailed in postmenopausal status (58.3%; 77/132), histological grade III (65.9%; 87/132), negative ER (50.8%; 67/132), Ki-67 ≥ 20% (75.8%; 100/132), negative HER2 (84.1%; 111/132), non-luminal A subtype (80.3%; 106/132), size T3/T4 (82.6%; 109/132), positive axilla (84.8%; 112/132) and stage III (80.3%; 106/132). Otherwise, carcinomas classified as Neo-Bioscore = 6–7 (9.0%; 28/310) prevailed in postmenopausal status (64.3%; 18/28), histological grade III (100%; 28/28), negative ER (75.0%; 21/28), Ki-67 ≥ 20% (92.9%; 26/28), negative HER2 (89.3%; 25/28), TN subtype (57.1%; 16/28), size T3/T4 (100%; 28/28), positive axilla (96.4%; 27/28) and stage III (100%; 28/28) (Table 1).

### Survival analysis

**Fig. 1:**
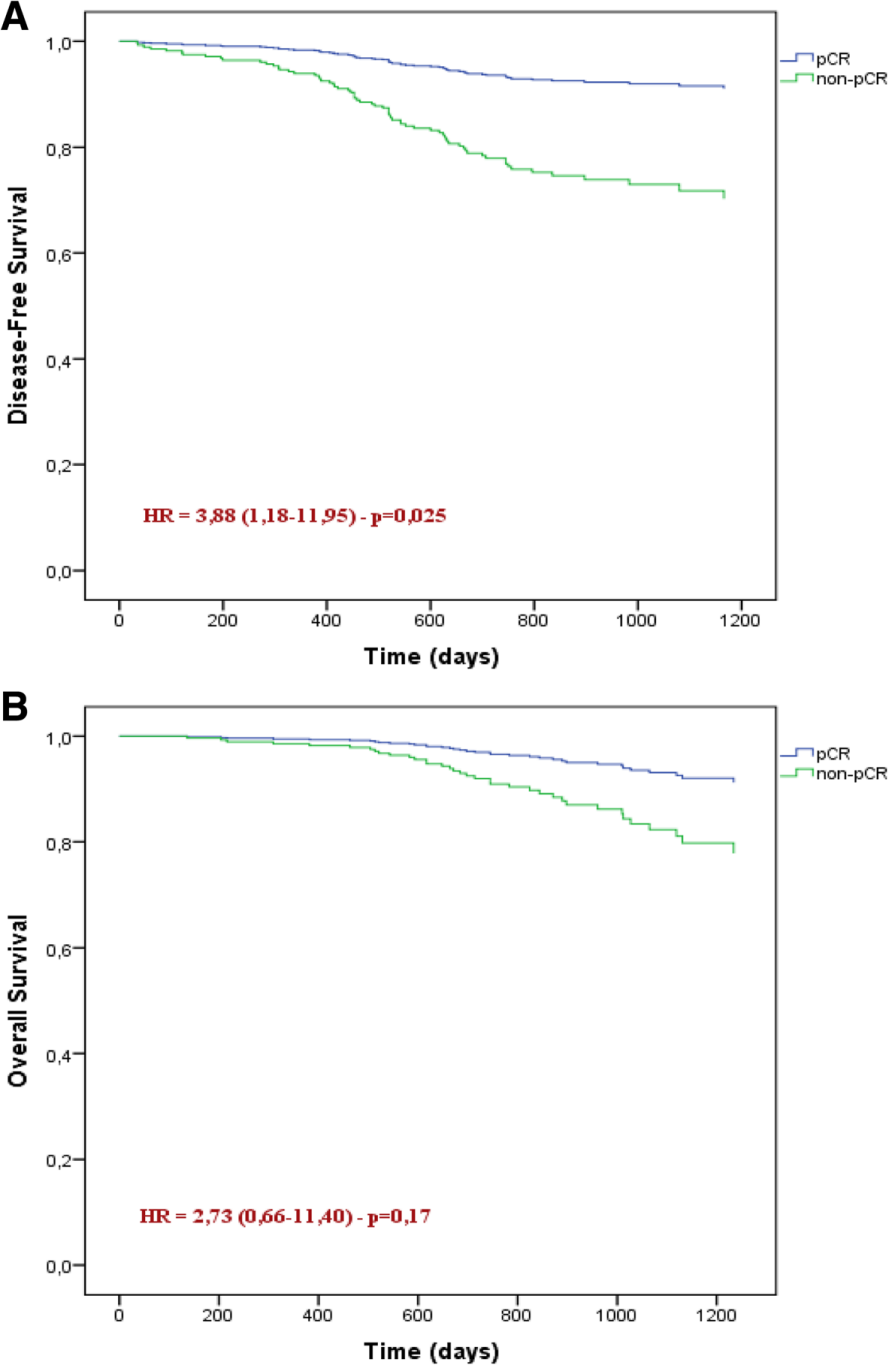
Disease-Free Survival (**a**) and Overall Survival (**b**) according to pathological response

The women enrolled in this study presented median time from first recurrence (mTFR) equal to 1274 d (CI95%: 1219–1329), DFS-3y = 0.75 (CI95%: 0.72–0.78), median overall survival (mOS) of 1405 d (CI95%: 1360–1449) and OS-3y = 0.81 (CI95%: 0.79–0.84). The pCR carcinoma occurred in 43 women (13.8%) with mTFR = 1432 d (CI95%: 1339–1525) and DFS-3y = 0.92 (CI95%: 0.88–0.96). Otherwise, 267 women had non-pCR carcinoma (86.7%) with mTFR = 1244 d (CI95%: 1182–1305) and DFS-3y = 0.69 (CI95%: 0.65–0.73) (Table 2). At 3-years non-pCR carcinoma showed a greater recurrence risk than pCR carcinoma with HR = 3.75 (CI95%: 1.18–11.95) but not the death risk with HR = 2.73 (CI95%: 0.66–11.35) (Fig. 1).

**Table 2:** Recurrence risk according to pathological response, Neo-Bioscore and Clustered Neo-Bioscore

Neo-Bioscore	All patients		pCR^c^		non-pCR	
	*n* = 310	DFS-3y (CI95%)^a^	*n* = 43	DFS-3y (CI95%)	*n* = 267	DFS-3y (CI95%)
0	1 (0.3%)	1.00	0	1.00	1 (0.4%)	1.00
1	18 (5.8%)	1.00	5 (11.6%)	1.00	13 (4.9%)	1.00
2	47 (15.2%)	0.88 (0.81–0.95)	12 (27.9%)	0.89 (0.79–0.99)	35 (13.1%)	0.86 (0.75–0.97)
3	84 (27.1%)	0.83 (0.78–0.88)	11 (25.6%)	0.91 (0.82–1.00)	73 (27.3%)	0.82 (0.76–0.88)
4	83 (26.8%)	0.78 (0.73–0.83)	14 (32.6%)	0.93 (0.87–1.00)	69 (25.8%)	0.74 (0.68–0.80)
5	49 (15.8%)	0.66 (0.57–0.75)	1 (2.3%)	1.00	48 (18.0%)	0.62 (0.51–0.73)
6	23 (7.4%)	0.13 (0.05–0.21)	0	–	23 (8.6%)	0.13 (0.05–0.21)
7	5 (1.6%)	0 (mTFR = 475 d)^b^	0	–	5 (1.9%)	0 (mTFR = 475 d)
Total		0.73 (0.70–0.76)	Total	0.92 (0.88–0.96)	Total	0.69 (0.65–0.73)
Clustered Neo-Bioscore	All patients		pCR		non-pCR	
	*n* = 310	DFS-3y (CI95%)	*n* = 43	DFS-3y (CI95%)	*n* = 267	DFS-3y (CI95%)
0–3	150	0.87 (0.83–0.91)	28 (65.1%)	0.93 (0.87–0.99)	122 (45.7%)	0.86 (0.82–0.90)
4–5	132	0.73 (0.68–0.78)	15 (34.9%)	0.92 (0.86–0.98)	117 (43.8%)	0.70 (0.65–0.75)
6	23 (7.4%)	0.13 (0.05–0.21)	0	–	23 (8.6%)	0.13 (0.05–0.21)
7	5 (1.6%)	0 (mTFR = 475 d)	0	–	5 (1.9%)	0 (mTFR = 475 d)

^a^*DFS-3y* 3-year disease-free survival rate

^b^*mTFR* median time to the first recurrence

^c^*pCR* pathological complete response

Considering the group of 43 pCR carcinomas, 65.1% of the total (28/43) was classified as Clustered Neo-Bioscore of low risk (Neo-Bioscore 0–3) with DFS-3y = 0.93 (0.87–0.99) and 34.9% (15/43) was of low-intermediate risk (Neo-Bioscore 4–5) with DFS-3y = 0.92 (0.86–0.98) (Table 2). For group of 267 non-pCR carcinomas, 45.7% (122/267) was of low risk with DFS-3y = 0.86 (CI95%: 0.82–0.90), 43.8% (117/267) was of low-intermediate risk with DFS-3y = 0.70 (CI95%: 0.65–0.75), 8.6% (23/267) was of high-intermediate risk (Neo-Bioscore 6) with DFS-3y = 0.13 (CI95%: 0.05–0.21) and 1.9% (5/267) was of high risk (Neo-Bioscore 7) with DFS-3y = 0 (mTFR = 475 d) (Table 2).

**Fig. 2:**
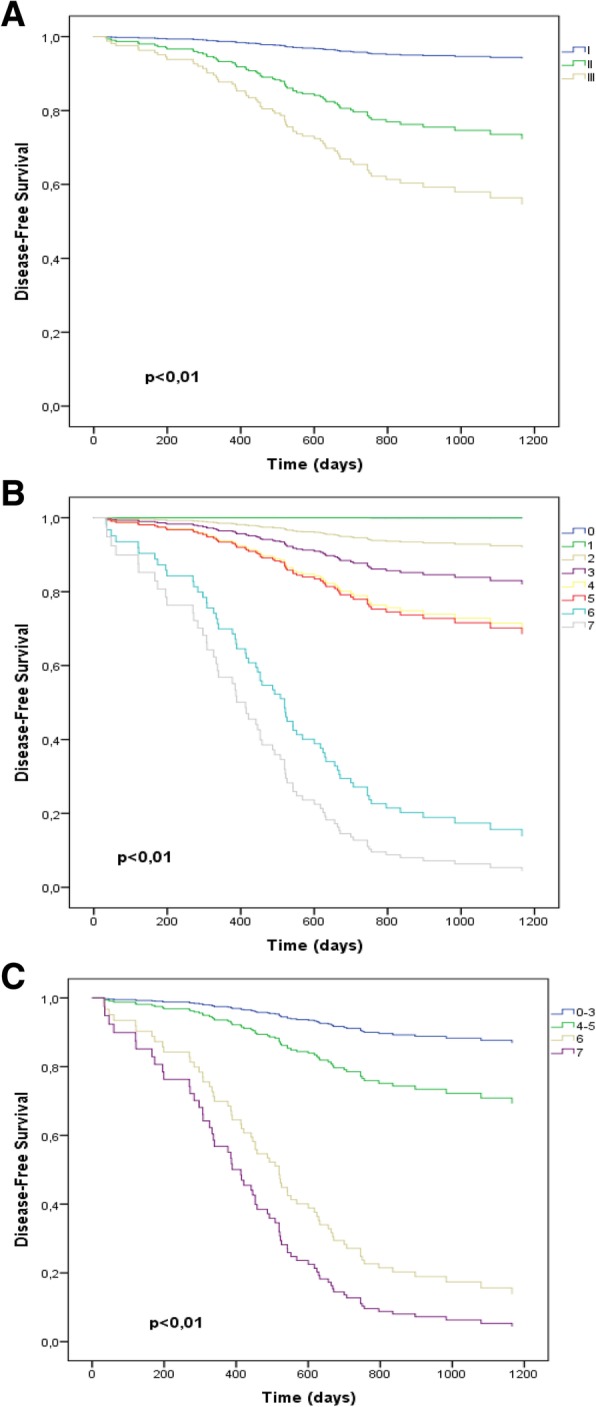
Disease-Free Survival for non-pCR carcinoma according to pathological stage (**a**), Neo-Bioscore (**b**) and Clustered Neo-Bioscore (**c**)

**Fig. 3:**
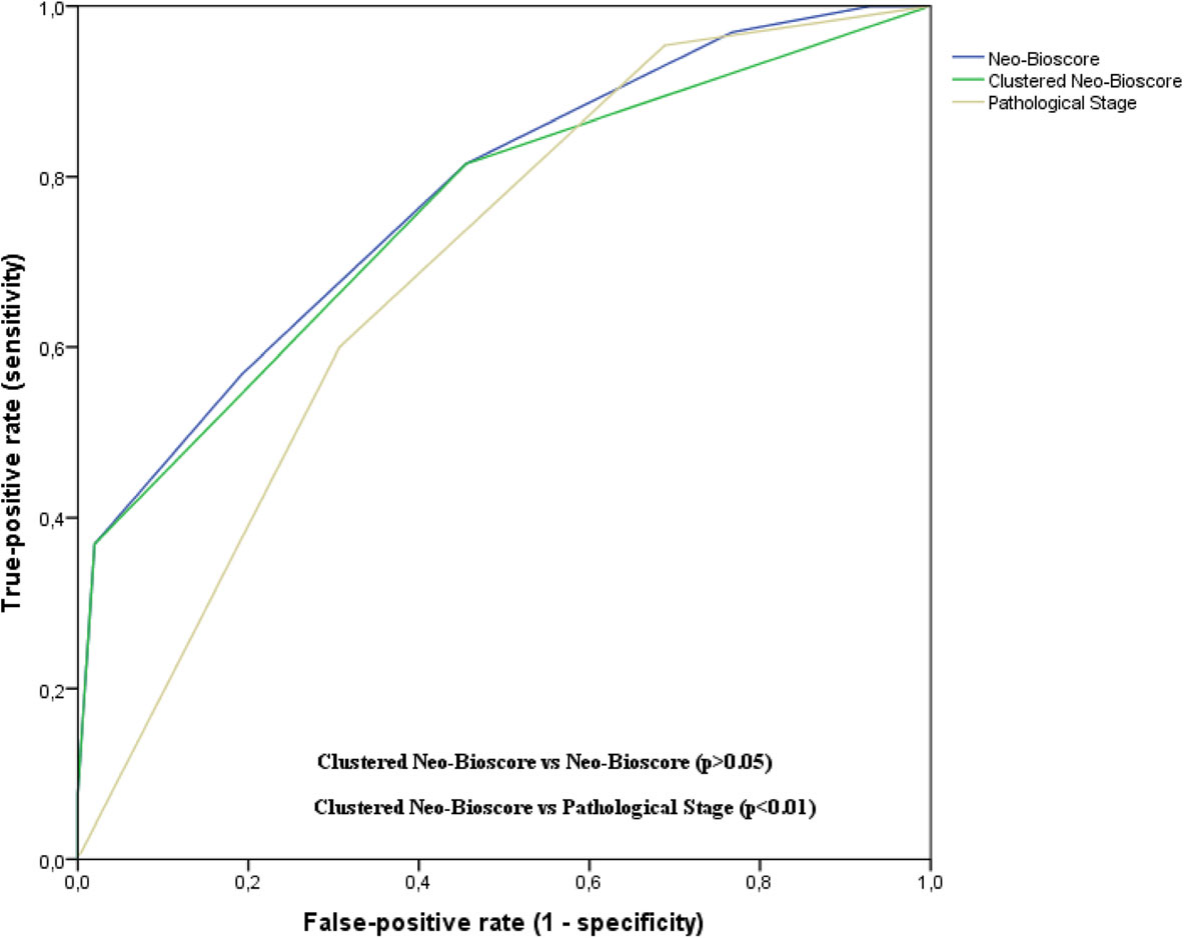
Prognostic accuracy by time-ROC method for non-pCR carcinoma according to pathological stage, Neo-Bioscore and Clustered Neo-Bioscore. AUC – Area Under Curve. AUC Clustered Neo-Bioscore = 0.76 (CI95%: 0.69–0.83). AUC Neo-Bioscore = 0.78 (CI95%: 0.71–0.84). AUC Pathological stage = 0.69 (CI95%: 0.63–0.76)

The subdivision of the scores for pathological staging (I, II and III), Neo-Bioscore (0 to 7) and Clustered Neo-Bioscore (low, low-intermediate, high-intermediate and high) presented statistical significance in relation to the risk of tumor recurrence for non-pCR carcinoma (*p* <  0.01) (Fig. 2). The prognostic accuracy was assessed by the time-ROC model (*p* > 0.05) between Neo-Bioscore (0.78, CI95%: 0.71–0.84) and Clustered Neo-Bioscore (0.76; CI95%: 0.69–0.83). Otherwise, these markers presented greater accuracy (p <  0.01) than the pathological stage (0.69; CI95%: 0.63–0.76) (Fig. 3).

The Clustered Neo-Bioscore subgroups of non-pCR carcinoma had remarkably different recurrence risks with statistical significance among them (p <  0.01). Considering the low risk as reference (mTFR = 1388 d), the low-intermediate risk presented mTFR = 1247 d (HR = 2.61, CI95%: 1.33–5.11), the high-intermediate risk had mTFR = 618 d (HR = 14.05, CI95%: 6.78–29.11) and the high risk showed mTFR = 433 d (HR = 22.19, CI95%: 7.70–63.92) (Table 3).

**Table 3:** Recurrence risk of non-pCR carcinoma according to Clustered Neo-Bioscore

Clustered Neo-Bioscore	mTFR^a^ (days)	*p*	HR^b^ (CI95%)
Low	1388	Reference	
Low-intermediate	1247	< 0.01	2.61 (1.33–5.11)
High-intermediate	618	< 0.01	14.05 (6.78–29.11)
High	433	< 0.01	22.19 (7.70–63.92)

^a^*mTFR* median time to the first recurrence

^b^*HR* Hazard ratio at 3 years

### Multivariate analysis

**Fig. 4:**
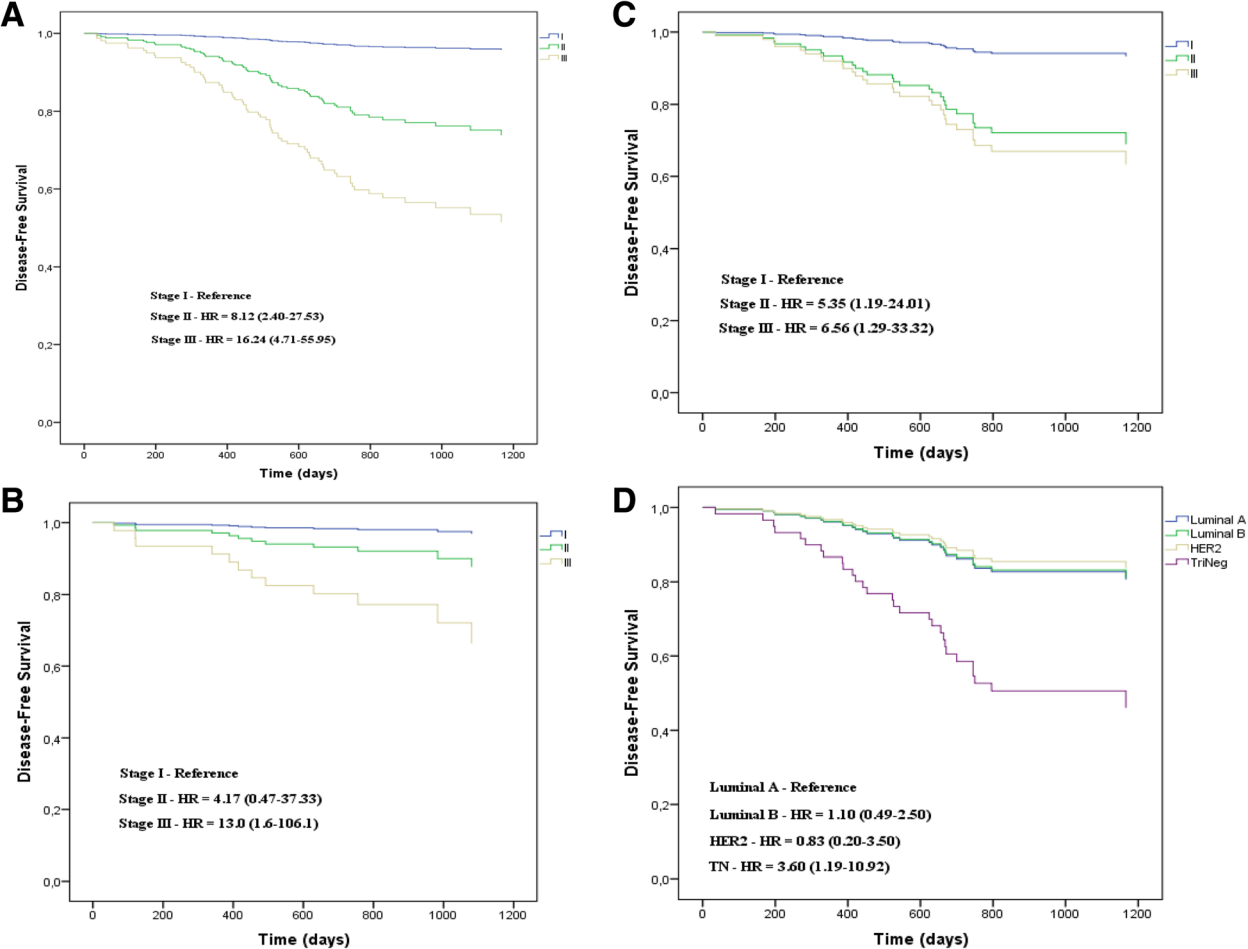
Independent prognostic factors of non-pCR carcinoma according to Neo-Bioscore (**a**), low (**b**) and low-intermediate Clustered Neo-Bioscore (**c**, **d**)

Considering Neo-Bioscore, the multivariate Cox regression analysis for tumor recurrence of non-pCR carcinoma showed that only the pathological stage was an independent prognostic factor. Taking the pathological stage I as the reference, the pathological stage II had HR = 8.12 (CI95%: 2.40–27.53) and the pathological stage III had HR = 16.24 (CI95%: 4.71–55.95). Otherwise, the Clustered Neo-Bioscore presented distinct results with greater discrimination of independent pathological factors compared to Neo-Bioscore. For the low risk, the pathological stage III was the only independent prognostic factor (HR = 13.0; CI95%: 1.6–106.1). For the low-intermediate risk, TN subtype (HR = 3.60; CI95%: 1.19–10.92) and pathological stages II (HR = 5.35; CI95%: 1.19–24.01) and III (HR = 6.56; CI95%: 1.29–33.32) were independent prognostic factors (Table 4 and Fig. 4).

**Table 4:** Recurrence risk by Cox regression analysis of non-pCR carcinoma according to Neo-Bioscore and Clustered Neo-Bioscore of low and low-intermediate risks

Characteristics	Neo-Bioscore	Low risk Clustered Neo-Bioscore	Low-intermediate Clustered Neo-Bioscore
	HR^a^ (CI95%)	HR (CI95%)	HR (CI95%)
Menopausal status			
Premenopausal	Reference	Reference	Reference
Postmenopausal	0.75 (0.45–1.27)	0.73 (0.19–2.77)	0.91 (0.41–2.03)
Histological grade			
I/II	Reference	Reference	Reference
III	1.33 (0.77–2.30)	2.05 (0.32–13.27)	0.77 (0.34–1.74)
Ki-67			
0–20%	Reference	Reference	Reference
20–50%	2.29 (0.68–7.73)	0.76 (0.12–4.79)	2.39 (0.29–19.89)
≥ 50%	3.39 (0.99–11.58)	4.81 (0.41–56.41)	3.37 (0.41–27.57)
Estrogen receptor			
Allred 7–8 (≥70%)	Reference	Reference	Reference
Allred 2–6 (< 70%)	1.54 (0.49–4.88)	2.17 (0.23–20.02)	0.53 (0.15–1.82)
Negative	1.35 (0.44–4.15)	1.33 (0.11–16.67)	1.39 (0.16–12.50)
HER2			
Negative	Reference	Reference	Reference
Positive	0.58 (0.15–2.28)	1.45 (0.34–6.17)	0.83 (0.20–3.50)
Tumor subtype			
A	Reference	Reference	Reference
B	0.50 (0.12–2.12)	0.53 (0.09–3.20)	1.10 (0.49–2.50)
HER2	0.58 (0.15–2.28)	1.45 (0.34–6.17)	0.83 (0.20–3.50)
TN	1.90 (0.49–7.30)	8.92 (0.94–84.93)	3.60 (1.19–10.92)
Clinical stage			
I/II	Reference	Reference	Reference
III	1.81 (0.88–3.75)	2.70 (0.60–12.21)	1.00 (0.38–2.66)
Pathological stage			
I	Reference	Reference	Reference
II	8.12 (2.40–27.53)	4.17 (0.47–37.33)	5.35 (1.19–24.01)
III	16.24 (4.71–55.95)	13.00 (1.60–106.10)	6.56 (1.29–33.32)

^a^*HR* Hazard ratio at 3 years

## Discussion

The Clustered Neo-Bioscore was a prognostic model of equivalent accuracy to Neo-Bioscore in women with non-pCR carcinoma but presented a more simplified categorization of scores, better differentiation among their prognostic scores and greater ability to identify independent prognostic factors.

Clustered Neo-Bioscore presented a prognostic accuracy in the risk of tumor recurrence with no statistically significant difference in relation to Neo-Bioscore for non-pCR breast carcinoma according to the ROC-time model. Otherwise, both models had greater prognostic accuracy compared than pathological stage [19, 20]. Neo-Bioscore previously showed greater prognostic accuracy than pathological staging but this analysis did not exclusively involve non-pCR breast carcinoma [21, 22]. Therefore, the clustering of risk scores of Neo-Bioscore based on prognostic similarity did not bring about a loss of prognostic power to Clustered Neo-Bioscore. On the other hand, Clustered Neo-Bioscore has a simpler pattern with only four risk scores to allocate and define the prognosis of women undergoing NAC who have a worse prognosis because they have residual carcinoma in surgical specimen.

Most published clinical trials have evaluated the prognostic factors of women undergoing NAC with pCR carcinoma, therefore, a better oncological prognosis [25–27]. Otherwise, this trial turned to a better understanding of the prognostic factors for women with non-pCR carcinoma due to their worse survival. The main objective of this trial was to assess the prognosis of women with residual carcinoma after NAC. Previous data show that usually 80–90% of the women submitted to NAC present residual carcinoma in the surgical specimen. Unlike most of the trials that evaluated pCR carcinoma this trial focused on the prognostic factors of women who did not reach pCR [19–22].

Analysis of survival results by Mittendorf’s pivotal trial [21] showed that the curves and survival at 3-year for 10-year follow-up were very similar to the results obtained in our trial. About 13.8% of the women had non-pCR carcinoma that showed a 3.88-fold greater risk of tumor recurrence than those with pCR carcinoma at 3-year follow-up (*p* = 0.025). However, there was no statistically significant difference for overall survival (*p* = 0.17) due to the shorter follow-up time since the survival curves between pCR and non-pCR carcinomas progressively move away from each other over time [23–26].

The subdivision of Clustered Neo-Bioscore into four risk scores was based on similar survival of Neo-Bioscore scores so that these new categories were independent and heterogeneous on prognosis. That is, the elevation of prognostic risk to tumor recurrence increased more linearly between the scores. Figure 2 showed that the survival curves for the risk scores of Clustered Neo-Bioscore were markedly distanced from each other, providing an adequate distinction of the prognostic value for tumor recurrence among the low, intermediate, high-intermediate, and high risk (*p* <  0.01). According to Table 3, non-pCR breast carcinoma classified as high-intermediate or high risk by Clustered-Neo-Bioscore, although only 9% of the total, presented a risk of recurrence much higher than those classified as low or low-intermediate, which account for about 91% of the total. Taking the low risk as a reference, high-intermediate and high risks had respectively 14-fold and 22-fold greater risk of tumor recurrence. Otherwise, women classified as low-intermediate risk had about 2.6-fold greater recurrence risk. Table 2 also showed that the distribution of risk scores in Clustered Neo-Bioscore at 3-years follow-up (DFS-3y) was more suitably heterogeneous to classify and distinguish women with non-pCR carcinoma. The confidence intervals of the DFS-3y values for the risk scores in Clustered Neo-Bioscore do not overlap unlike the Neo-Bioscore scores of 0 to 5.

Considering the Neo-Bioscore model, Cox regression showed that only the pathological stage was an independent prognostic factor for tumor recurrence in women with non-pCR breast carcinoma after NAC. The results showed that stages II and III were associated with 8-fold and 16-fold greater recurrence risk compared to the stage I. On the other hand, Neo-Bioscore did not identify any biological marker as a prognostic risk, contrary to what has been observed in some trials in which the biological nature of the tumor plays an important role in the prognosis [23, 25–28, 43]. Regarding Clustered Neo-Bioscore, Cox regression can only be performed for non-pCR carcinomas of low and low-intermediate risks which accounted for about 91% of total women. Non-pCR carcinomas of high-intermediate and high risks enrolled only 23 and 5 patients, respectively, which made it impossible to perform the multivariate analysis.

Clustered Neo-Bioscore presented Cox regression results different from Neo-Bioscore with better power in discriminating independent prognostic factors. For non-pCR carcinoma of low risk, only pathologic stage III was an independent prognostic factor with 13-fold greater recurrence risk compared to the stage I. That is, only bulky tumor after NAC (greater than 5 cm and/or N2/N3) had worse prognosis in low-risk non-pCR carcinoma. Otherwise, non-pCR carcinoma of low-intermediate risk showed that TN subtype (3.60-fold) and pathological stages II (5.35-fold) and III (6.56-fold) had a greater risk of tumor recurrence. Some clinical trials have shown the prognostic risk of residual tumor after NAC but there is less information on the prognostic correlation between residual tumor volume and the biological factors of carcinoma as shown by the low-intermediate risk [24, 28, 43]. TN carcinoma is usually highly chemosensitivity and has high potential to reach pCR. However, results for non-pCR carcinoma of low-intermediate risk showed that TN subtype has a worse prognosis when it is associated with residual tumor after NAC (stages II and III) [16–22, 27, 43].

The results provided by Clustered Neo-Bioscore indicate the need to better identify prognostic factors associated with non-PCR breast cancer to evaluate new strategies related to NAC such as the number of chemotherapy cycles to be used, drug combination, assessment of tumor downstaging, identification of new biomarkers of tumor resistance or new protocols to complement the NAC in order to reduce the risk of tumor recurrence. The breast carcinomas analyzed in this clinical trial presented high-risk clinical and biological characteristics predominating size T3/T4 size, positive axilla, clinical stage III, histological grade III, non-luminal carcinoma A, negative ER and Ki-67 ≥ 20%.

## Conclusions

In summary, Clustered Neo-Bioscore demonstrated potential as a better marker for prognostic analysis of non-pCR carcinoma undergoing NAC, since it had a prognostic accuracy equivalent to Neo-Bioscore, but presented a more simplified format in the subdivision of risk scores, a better model to discriminate the prognosis among the risk scores due to the greater prognostic differentiation and a better pattern in the assessment of independent prognostic factors in women classified as low and low-intermediate risks.

This is a retrospective clinical trial with limitations inherent to bias control and confounding factors, but we believe that it makes an important scientific contribution to understanding the prognostic factors of non-pCR breast carcinoma. External validation trials should be performed to evaluate the generality of the results obtained.

## Additional file

Additional file 1: Datasets supporting the study findings. (XLSX 122 kb)

## Data Availability

The dataset supporting the conclusions of this article is included as an Additional file 1.

## Abbreviations

AC: Doxorubicin plus cyclophosphamide
AUC: Area under the curve
CAISM: Woman’s Hospital Dr. José Aristodemo Pinotti
DFS: Disease-free survival
ER: Estrogen receptor
FCM: Faculty of Medical Sciences
HER2: Human epidermal growth factor
HR: Hazard ratio
Ki-67: Cellular proliferation index
mOS: Median overall survival
mTFR: Median time from first recurrence
NAC: Neoadjuvant chemotherapy
non-pCR: Pathological non-complete response
OS: Overall survival
pCR: Pathological complete response
PR: Progesterone receptor
ROC: Receiver operating characteristic
T: Paclitaxel
TN: Triple-negative
UNICAMP: State University of Campinas
